# Supplementary material for: Absorption rate of subcutaneously infused fluid in ill multimorbid older patients
Source: PLoS One. 2022 Oct 10;17(10):e0275783. doi: 10.1371/journal.pone.0275783 (PMC9550057; doi:10.1371/journal.pone.0275783)
Supplement: S1 File — (PDF) [file pone.0275783.s001.pdf]

Supporting information to

Absorption Rate of Subcutaneously Infused Fluid in Ill Multimorbid Older Patients

Contents

S1 Fig. Mixed logistic regression with quadratic effect on fluid remaining in the SC space..... 1

S2 Fig. Mixed logistic regression with quadratic effect on absorption based on data from the blood samples........ 2

S3 Fig. Mixed logistic regression with quadratic effect on absorption based on data from the thyroid gland........ 3

S1 Fig. Mixed logistic regression with quadratic effect on fluid remaining in the SC space

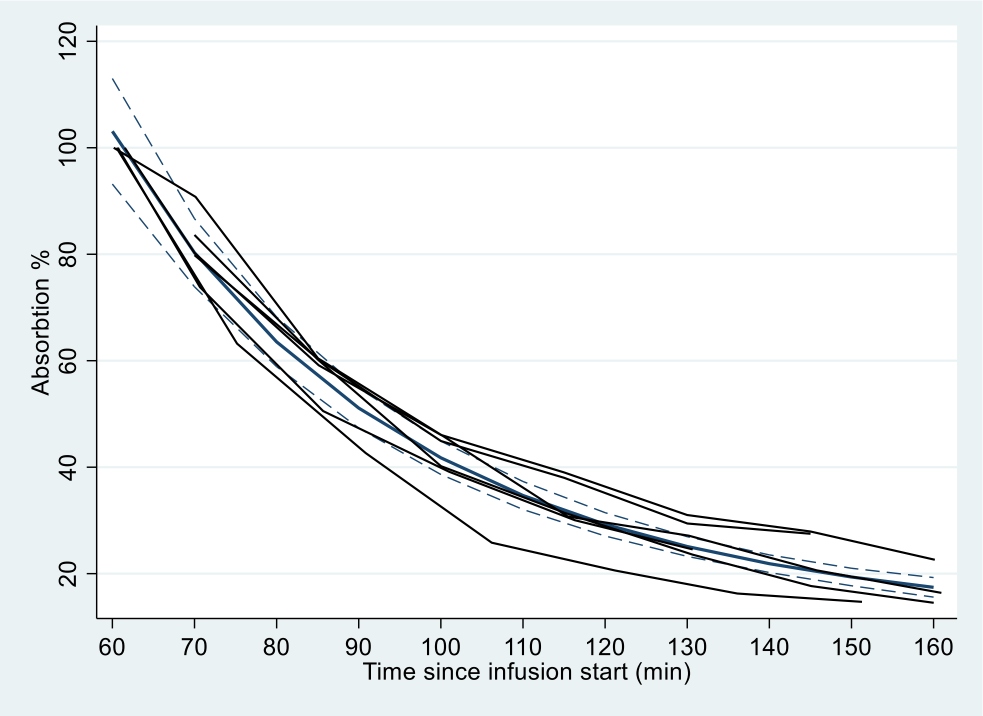

Change in activity at the infusion site after the end of infusion (60 minutes). The y-axis is percentage of maximum activity. The blue line is the fitted curve, the dashed lines are the 95% confidence for the fitted line, and the gray lines are the actual measurements from our patients.

S2 Fig. Mixed logistic regression with quadratic effect on absorption based on data from the blood samples

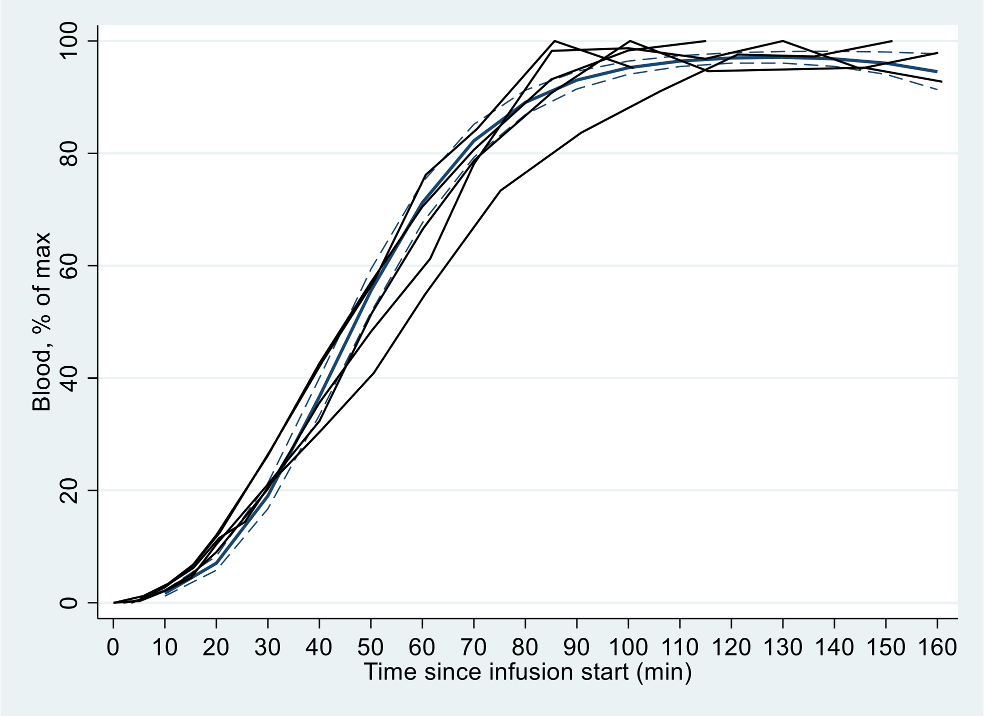

Change in activity in the blood. The y-axis is percentage of maximum activity. The blue line is the fitted curve, the dashed lines are the 95% confidence for the fitted line, and the gray lines are the actual measurements from our patients.

S3 Fig. Mixed logistic regression with quadratic effect on absorption based on data from the thyroid gland

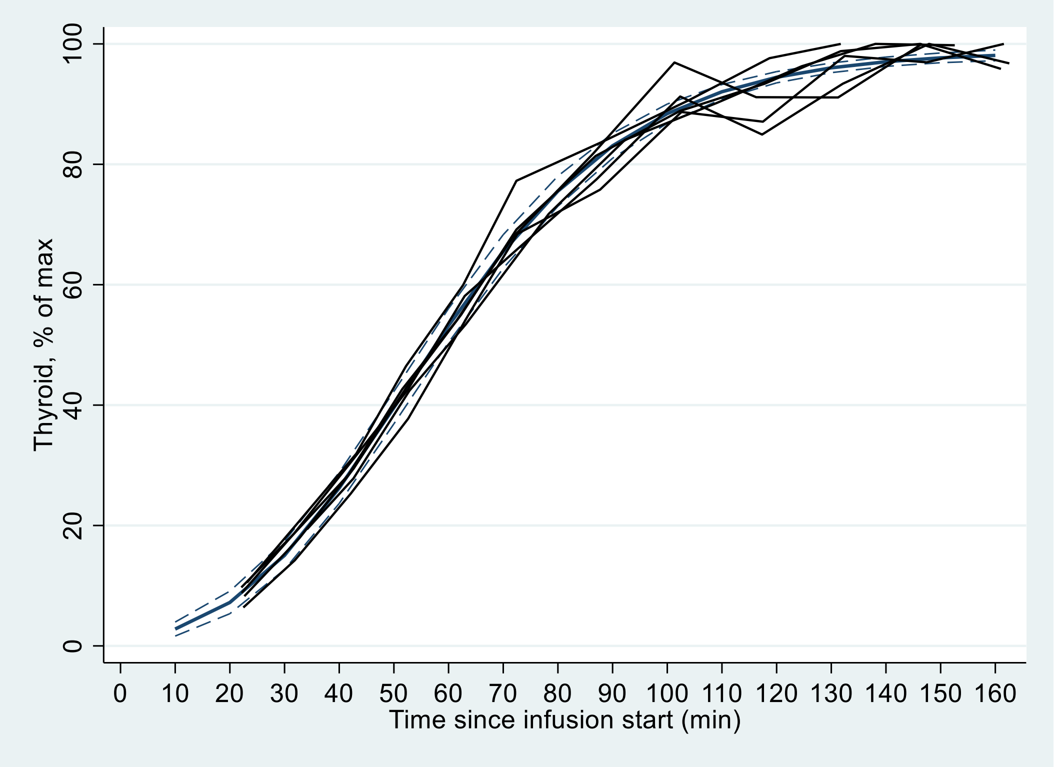

Change in activity in the thyroid gland. The y-axis is percentage of maximum activity. The blue line is the fitted curve, the dashed lines are the 95% confidence for the fitted line, and the gray lines are the actual measurements from our patients.
