# Supplementary material for: Absorption rate of subcutaneously infused fluid in ill multimorbid older patients
Source: PLoS One. 2022 Oct 10;17(10):e0275783. doi: 10.1371/journal.pone.0275783 (PMC9550057; doi:10.1371/journal.pone.0275783)
Supplement: S1 Protocol — (PDF) [file pone.0275783.s002.pdf]

1. Den originale titel

Absorption rate of subcutaneous infused fluid.

Dansk titel:

Forskningsprojektet: Optagelse af væske givet i underhuden på maven

2. Formål

Formålet med studiet er, at undersøge og beskrive hvor hurtigt subkutant infunderet væske absorberes, og er tilgængelig for kredsløbet hos akut syge ældre patienter i forhold til ikke akut syge ældre. Ved akut syge menes patienter der er indlagt akut og aktuelt behandles for en akut tilstand. Ved "ikke akut syge" menes patienter der ikke aktuelt behandles for en akut sygdom, men godt kan have kroniske sygdomme i stabil fase. Vores hypotese er, at væske absorberes langsommere hos den akut syge end hos den raske. Subkutan væskeindgift bruges på ældre patienter med behov for parenteralt væsketilskud. Vejledninger beskriver, at det kun må bruges på stabile patienter[1]. Ved at bestemme absorptionshastigheden og hvor hurtigt væsken er tilgængelig for kredsløbet, kan patientgruppen, hvor subkutan væskeinfusion er indiceret, præciseres og muligvis udvides. Der er også kommet øget fokus på subkutan medicinering[2], og dette studie kan øge kendskabet til de ændringer, der sker i den ældres krop under akut sygdom.

Baggrund:

Formålet med parenteral hydrering er at tilføre væske til kredsløbet. Ved intravenøs hydrering er dette opnået, men ved subkutan hydrering skal væsken først absorberes fra det subkutane rum til karbanen for at opnå den ønskede effekt.

For relevant at kunne evaluere effekten af subkutan hydrering, bør vi kende absorptionshastigheden. Lipschitz et al. undersøgte i 1991 absorptionshastighed og fandt, at væsken var tilgængelig inden for en time. Deres studie var dog gjort på raske +65-årige og med tilføjelse af hyaluronidase (der øger absorptionshastigheden og som ikke benyttes længere.)[3]

Ved subkutan infusion af en krystalloid opløsning absorberes det meste af væsken gennem passiv diffusion til kapillærerne,[4] men ved akut sygdom øges lækket fra kapillærerne.[5] Det øgede læk kan potentielt nedsætte absorptionshastighed af væsken og dermed forlænge tid fra infusion til effekt i kredsløbet. Alternativt kan den akutte sygdom nedsætte modstanden i det subkutane rum og dermed øget absorptionsoverfladen, hvorved absorptionshastigheden øges.

Radioaktive markører kan bruges til både at vurdere hastigheden af absorption fra subkutis (fald i radioaktivitet i subkutis) og tilstedeværelsen i karbanen (målbar radioaktivitet i blodprøver). Lipschitz et al. brugte både pertechnetat (TcO_4^-) og tritieret vand og viste at pertechnetat havde samme absorptionshastighed som tritieret vand.[3]

3. Metode

Forsøget er et case-kontrol studie med forsøgsdeltager som deres egen kontroller. Vi vil rekruttere geriatriske patienter aktuelt indlagt. Vi vil inviteret patienterne tilbage 8 uger efter udskrivelse for, at undersøge om der er forskel imellem akut syg og ikke akut syg (stabil fase). Vi forventer, at kun en del af inkluderede patienter ønsker / kan deltage 8 uger efter udskrivelse.

Patient flow:

1. Stuegangsgående læger vurderer, om nogle af de indlagte patienter er relevante at inkludere.
2. Mundtlig og skriftlig information til relevante patienter mens deres pårørende er tilstede.
3. 24-48 timers betænkningstid.
4. Underskrift af samtykkeerklæring.
5. Patienterne deltager i første undersøgelse (absorption under akut sygdom).

   <u>Undersøgelsen (ens for første og anden gang):</u>
6. 9:00: Patienten ankommer til forsøgslokalet (køres af portør).
   Forsøgets indhold for patienten gennemgås igen.
7. 9:15: Anlæggelse af subkutan nål i maveskindet og blodprøvetagningsnål i en stor vene (v. cubiti).
8. 9:30-10:30: Infusion af 250 ml NaCl hvor 30 MBq pertechnetat er opblandet i.
9. 9:30-15:00: Detektion af gammaaktivitet over infusionsstedet og i udtaget blod.

10. 3-6 uger efter patienterne er udskrevet fra hospitalet, kontakter vi dem igen mhp. at finde en dato til anden undersøgelse.
11. Anden undersøgelse følger samme metode som den første (punkt 6-9).

Subkutan nål er: "BD Saf-T-Intima™ GA22 - Integrated Safety Catheter System". Blodprøver under forsøget vil blive taget ved at anlægge en heparineret venflon i v. cubiti.

4. Statistiske overvejelser

Lipschitz et al. beskrev i deres tekniske artikel absorptionshastigheden ved en absorptionskonstant fundet ved nonlinear regression[6]. De fandt en absorptionskonstant på 2,29 time^{-1} for subkutan infusion med hyaluronidase men beskriver ingen værdier uden hyaluronidase. Hvis vi antager en forskel på 15% mellem akut syg ikke akut syg (vi har intet data at basere dette på, så dette er et skøn) og bruger 2,29 time^{-1} som ikke akut syg værdi, vil den gennemsnitlig forskel (effektstørrelse) være 0,345 time^{-1}. Standardafvigelse baserer vi fra Lipschitz første infusion i hver patient hvilket var = 0,3 time^{-1}. Med en alpha på 0,05 og beta på 0,80 får vi en gruppe størrelse på 6 (paired T-test).

Da vi forventer at en stor del af patienterne ikke har lyst eller kræfter til at gennemføre anden undersøgelse forventer vi at skulle rekruttere ca. 15 deltager.

5. Forsøgspersonerne

Inklusionskriterier:

- Geriatrisk patient
- +75 år
- Evne til at afgive informeret samtykke.

Eksklusionskriterier:

- Væskerestriktion
- Svært syg med risiko for akut forværring.
- Patienter hvor det vurderes af læge eller plejepersonale at det vil være uhensigtsmæssigt at de opholder sig væk fra sengeafdeling i de timer forsøget tager.
- Vurderet kort restlevetid.

6. Risici, bivirkninger og ulemper på kort og lang sigt

Ved anlæggelse af både den subkutane nål og blodprøvetagningsnål kan der være mindre gener i form af nålestikssmerter ved anlæggelse. Der kan opstå et mindre hæmatom (blåt mærke) ved indstiksstedet. Indgift af subkutan væske forventes ikke at påføre patienterne nogle gener da vi kun giver en relativt lille volumen, og nålen kun ligger i et par timer[7]. Der kan forekomme lidt svie, som afhjælpes ved at sænke infusionshastigheden. Der kan være uforudsete risici og belastninger forbundet med forsøget. Patienterne må gerne medbringe pårørende på forsøgsdagen hvis de ønsker.

Stråledosis til kroppen som helhed (effektiv dosis) ved intravenøs indgift (ingen data på subkutan indgift) af pertechnetat er 0,013 mSv/MBq (table C.87; Intravenous administration, no blocking agent given)[8]. Ved indgift på 30 MBq pertechnetat vil effektiv dosis vil være ca. 0,8 mSv for begge dage tilsammen (2 × 30 MBq × 0,013 mSv/MBq = 0,78 mSv), det halve hvis patienten kun deltager på første forsøgsdag. For en gennemsnitsperson svarer dette til en øget risiko for stokastisk skade på 0,004% (0,002% ved deltagelse på kun første forsøgsdag). Da vores patienter er blandt de ældste forsøgspersoner, er risikoen for en stokastisk skade mindsket med en faktor 5-10 (jævnfør Retningslinjer om anvendelse af ioniserende stråling i sundhedsvidenskabelige forsøg, Appendiks 2, 2011, NVK).

Overvejelser om subkutan indgift: Ovenstående tal var for intravenøs indgift, mens der ikke umiddelbar findes data på subkutan indgift. Da bestråling aftager hurtigt med afstanden (afstandskvadratloven), vil den væsentligste bestråling af et organ være når stoffet er i umiddelbar nærhed af organet, såsom i blodtilførslen til organet. For kroppen som helhed vil bestrålingen derfor væsentligst ske efter at pertechnetat har nået blodbanen, dvs. ud fra ovenstående beregning. Vævet på selve injektionsstedet vil få en større stråledosis end kroppen som helhed, men hud og muskler hører ikke til de specielt strålefølsomme dele af kroppen. Til sammenligning er grænserne for stråleudsatte arbejdstagere 25 gange større for hud og ekstremiteter (500 mSv/år) end for kroppen som helhed (20 mSv/år). Ref: Bekendtgørelse nr. 669 om ioniserende stråling og strålebeskyttelse (Strålebeskyttelsesbekendtgørelsen), bilag 1. https://www.retsinformation.dk/Forms/R0710.aspx?id=209405

7. Udtagning af nyt biologisk materiale eller indsamling af biologisk materiale fra allerede eksisterende biobank

Under forsøget udtages blod løbende fra patienterne. Vi vil udtage blod efter 5, 10, 15, 30, 45, 60, 75, 90, 105, 120, 150, 180 min. efter start af infusion. Vi vil udtage 5 ml per gang. Vi vil måle aktivitet i det udtagne blod samme dag (eller evt. efterfølgende dag) og derefter destruere det. Det udtagende blod bruges til at vurderer hvor hurtigt den infunderede væske findes i blodbanen. Intet blod eller andet biologisk materiale gemmes i en biobank.

Ved deltagelse på anden forsøgsdag vil patienterne få taget blodprøver til måling af CRP, Hb, leu, Na, K, Glu, Osml, Krea, Karb, Alb før forsøges startes. Disse vil blive analyseret som vanligt. De samme blodprøver vil blive trukket fra patienternes journal i forbindelse med den første undersøgelse, hvorfor der ikke er behov for at tage dem.

8. Oplysninger fra patientjournaler

Stuegangslæger vil vurdere indlagte patienter og diskutere med forsøgsansvarlig vedrørende hvorvidt patienten opfylder inklusion- eller eksklusionskrieteriere. Relevante kandidater vil blive kontaktet af forsøgsansvarlig, og vi vil finde et tidspunkt hvor det passer dem og deres pårørende at gennemgå informationer vedrørende forsøget samt samtykke erklæring. Hvis patienterne ønsket at deltage vil følgende informationer blive trukket fra deres journal: Alder, køn, indlæggelsesårsag, diagnose liste, aktuel medicinering, blodtryk, Puls, saturation, respirations frekvens, blodprøver (CRP, Hb, leu, Na, K, Glu, Osml, Krea, Karb, Alb). Forsøgsansvarlig vil som følge af informeret samtykke kunne indhente oplysninger direkte fra patientens journal. Disse oplysninger kan videregives til relevant tilsynsmyndighed som led i kvalitetskontrol og eller monitorering.

Ingen informationer fra patienternes journal vil blive videregivet til forsøgsansvarlige (fraset navn og evt. stue nummer) før informeret samtykke er givet, dog kan forsøgsansvarlig ved tvivl om patienterne opfylder eksklusionskriterier vurdere dette bland andet ved hjælp af journal notater.

9. Behandling af personoplysninger i projektet

Alt data vil blive opbevaret sikkert i REDCap, og databeskyttelsesforordningen og -loven overholdes jf. komitélovens § 20, stk. 1, nr. 4. Projektet er ansøgt og godkendt af Datatilsynet gennem Region Nordjyllands paraply-ansøgning.

Efter forsøget vil data blive gemt efter reglerne for "Danish Code of Conduct for Research Integrity". Originale data vil evt. blive delt i anonymiseret form med andre forskere efter kontakt til forsøgsansvarlig hvis formålet findes relevant, f.eks. kontrol af resultater, statistisk metode eller ny behandling af data.

10. Økonomi

Projektet er initieret og sponsoreret af Geriatrisk afdeling, Aalborg Universitetshospital. Ydermere er der øget tilskud til projektet fra Endokrinologisk afdeling og Nuklearmedicinsk afdeling i form af materiale og personaletid.

11. Evt. vederlag og/eller andre goder til forsøgspersonerne

Forsøgspersoner vil ikke modtage vederlag.

12. Rekruttering af forsøgspersoner og informeret samtykke

Stuegangsgående læger vil vurdere om nogle af de indlagte patienter er relevante til inklusion i projektet. Disse vil blive kontaktet af forsøgsansvarlig, få udleveret skriftlig deltagerinformation og sammen finde en tid hvor de og deres pårørende (hvis de ønsker at disse skal deltage) kan mødes i et mødelokale. Her vil de modtage mundtlig deltagerinformation fra forsøgsansvarlig. Efterfølgende vil de have 24-48 timers betænkningstid, før de evt. underskriver samtykkeerklæringen.

Deltager vil på hvilket som helt tidspunkt kunne trække deres samtykke tilbage, uden dette vil have konsekvens for deres videre behandling.

Når patienterne er udskrevet fra hospitalet, vil vi kontakte dem 3-6 uger efter for at finde tid til undersøgelse nummer 2.

13. Offentliggørelse af resultater

Resultatet vil blive forsøgt offentliggjort i relevante tidsskrifter uafhængig af om resultatet er positivt, negativt eller inkonklusivt. Ydermere vil forsøget blive registreret på clinicaltrials.gov eller anden officiel registreringsside hvor resultaterne vil blive offentliggjort hvis ikke de kan blive offentliggjort i et tidsskrift.

14. Videnskabsetisk afsnit

Subkutan væskeindgift er en infusionsmetode med lille risiko for bivirkninger og anlæggelsen af nålen er uden særlig gene for patienterne. Den stråle relaterede risiko er lille, specielt når de inkluderede patienters alder tages i betragtning. Vi vurderer derfor at den samlede risikoen for de inkluderede patienter er lille og selvom der ikke er en sundhedsgevinst for de inkluderede patienter, vurderer vi at den samlede videnskabelige og kommende sundhedsmæssige gevinst retfærdiggør risikoen og besværet.

15. Oplysninger om erstatningsordning

Projektet er omfattet af patienterstatningen.

1. Thomas DR, Cote TR, Lawhorne L, et al. Understanding Clinical Dehydration and Its Treatment. *J Am Med Dir Assoc*. 2008;9(5):292-301.

2. Noriega OD, Yarlequé León SN. Antibiotics by Subcutaneous Route: A Safe and Efficient Alternative. *J Am Med Dir Assoc*. 2018;19(6):553-554.

3. Lipschitz S, Campbell AJ, Roberts MS, et al. Subcutaneous fluid administration in elderly subjects: validation of an under-used technique. *J Am Geriatr Soc*. 1991;39(1):6-9.

4. STONE PW, MILLER WB. Mobilization of radioactive sodium from the gastronomies muscle of the dog. *Proc Soc Exp Biol Med*. 1949;71(4):529-534.

5. Cordemans C, De laet I, Van Regenmortel N, et al. Fluid management in critically ill patients: the role of extravascular lung water, abdominal hypertension, capillary leak, and fluid balance. *Ann Intensive Care*. 2012;2(Suppl 1):S1.

6. Roberts MS, Lipschitz S, Campbell AJ, Wanwimolruk S, McQueen EG, McQueen M. Modeling of subcutaneous absorption kinetics of infusion solutions in the elderly using technetium. *J Pharmacokinet Biopharm*. 1997;25(1):1-21.

7. Caccialanza R, Constans T, Cotogni P, Zaloga GP, Pontes-Arruda A. Subcutaneous Infusion of Fluids for Hydration or Nutrition: A Review. *J Parenter Enter Nutr*. 2018;42(2):296-307.

8. Mattsson S, Johansson L, Leide Svegborn S, et al. ICRP Publication 128: Radiation Dose to Patients from Radiopharmaceuticals: a Compendium of Current Information Related to Frequently Used Substances. *Ann ICRP*. 2015;44(2_suppl):7-321.
