# Supplementary material for: Absorption rate of subcutaneously infused fluid in ill multimorbid older patients
Source: PLoS One. 2022 Oct 10;17(10):e0275783. doi: 10.1371/journal.pone.0275783 (PMC9550057; doi:10.1371/journal.pone.0275783)
Supplement: S2 Protocol — (PDF) [file pone.0275783.s003.pdf]

Title:

Uptake of fluid given into the subcutaneous tissue of the stomach

2. Purpose

The purpose of the study is to investigate and describe how quickly subcutaneous infused fluid is absorbed, and is available to the circulation in acutely ill elderly patients compared to non-acutely ill elderly. By "acutely ill" is meant patients who have been admitted acutely and are currently being treated for an acute condition. By "not acutely ill" is meant patients who are not currently being treated for an acute illness, but may well have chronic diseases in a stable phase. Our hypothesis is that fluid is absorbed more slowly in the acutely ill than in the healthy. Subcutaneous fluid administration is used in elderly patients in need of parenteral fluid supplementation. Guidelines describe that it should only be used on stable patients [1]. By determining the rate of absorption and how quickly the fluid is available to the circulation, the patient group in which subcutaneous fluid infusion is indicated can be clarified and possibly expanded. There has also been an increased focus on subcutaneous medication [2], and this study may increase awareness of the changes that occur in the elderly person's body during acute illness.

Background:

The purpose of parenteral hydration is to supply fluid to the circulation. In intravenous hydration, this is achieved, but in subcutaneous hydration, the fluid must first be absorbed from the subcutaneous space to the circulation to achieve the desired effect.

To be able to relevantly evaluate the effect of subcutaneous hydration, we should know the rate of absorption. Lipschitz et al. examined in 1991 absorption rate and found that the liquid was available within an hour. However, their study was performed on healthy + 65-year-olds and with the addition of hyaluronidase (which increases the rate of absorption and is no longer used.) [3]

By subcutaneous infusion of a crystalloid solution, most of the fluid is absorbed through passive diffusion to the capillaries, [4] but in acute disease, the leakage from the capillaries increases. [5] The increased leak can potentially decrease the rate of absorption of the liquid, thereby prolonging the infusion time from the power in the circuit. Alternatively, the acute disease may decrease the resistance in the subcutaneous space and thus increase the absorption surface, thereby increasing the rate of absorption.

Radioactive markers can be used to assess both the rate of absorption from the subcutis (decrease in radioactivity in the subcutis) and the presence in the circulation (measurable radioactivity in blood samples). Lipschitz et al. used both pertechnetate (TcO_4^-) and tritiated water and showed that pertechnetate had the same rate of absorption as tritiated water. [3]

3. Method

The trial is a case-control study with trial participants as their own controls. We will recruit geriatric patients currently hospitalized. We will invite patients back 8 weeks after discharge to investigate whether there is a difference between acutely ill and not acutely ill (stable phase). We expect that only some of the included patients want / can participate 8 weeks after discharge.

Patient flow:

1. Inpatient physicians assess whether some of the hospitalized patients are relevant to include.
2. Oral and written information is given to relevant patients while their relatives are present.
3. 24-48 hours reflection time.
4. Signing of consent form.
5. Patients participate in the first study (absorption during acute illness).

The examination (same for the first and second time):

6. 9:00: The patient arrives at the experimental room.

   The content of the trial for the patient is reviewed again.

7. 9:15: Insertion of subcutaneous needle into abdominal skin and blood sampling needle into a large vein (v. Cubiti).
8. 9: 30-10: 30: Infusion of 250 ml NaCl with 30 MBq pertechnetate mixed in.
9. 9: 30-15: 00: Detection of gamma activity over the infusion site and in drawn blood.

10. 3-6 weeks after the patients have been discharged from the hospital, we contact them again for to find a date for another study.
11. The second study follows the same method as the first (points 6-9).

The subcutaneous needle is: “BD Saf-T-Intima ™ GA22 - Integrated Safety Catheter System”. Blood samples taken during the experiment will be taken by applying a heparinized venflon in the v. Cubiti.

4. Statistical considerations

Lipschitz et al. described in their technical article the rate of absorption by an absorption constant found by nonlinear regression [6]. They found an absorption constant of 2.29 h^{-1} for subcutaneous infusion with hyaluronidase but do not describe values without hyaluronidase. If we assume a difference of 15% between acutely ill not acutely ill (we have no data to base this on, so this is an

estimate) and use 2.29 hours^{-1} as non acutely ill value. We base the standard deviation from Lipschitz first infusion in each patient which was = 0.3 hour^{-1}. With an alpha of 0.05 and a beta of 0.80, we get a group size of 6 (paired T-test).

As we expect that a large proportion of patients do not have the desire or energy to carry out the second examination, we expect to have to recruit approx. 15 participants.

5. The subjects

Inclusion criteria:

- Geriatric patient
- +75 years
- Ability to provide informed consent.

Exclusion criteria:

- Fluid restriction
- Severely ill with risk of acute exacerbation.
- Patients where it is assessed by a doctor or nursing staff that it would be inappropriate for them to stay away from the ward for the hours the examination takes.
- Estimated short remaining life.

6. Risks, side effects and disadvantages in the short and long term

When applying both the subcutaneous needle and the blood sampling needle, there may be minor inconvenience in the form of needle pain during application. A small hematoma (bruise) may occur at the injection site. Administration of subcutaneous fluid is not expected to cause any inconvenience to patients as we only give a relatively small volume and the needle is only left for a few hours [7]. There may be some burning, which is relieved by slowing down the infusion rate. There may be unforeseen risks and strains associated with the trial. Patients are welcome to bring relatives on the day of the trial if they wish.

The radiation dose to the body as a whole (effective dose) for intravenous administration (no data on subcutaneous administration) of pertechnetate is 0.013 mSv / MBq (Table C.87; Intravenous administration, no blocking agent given) [8]. At administration of 30 MBq pertechnetate, the effective dose will be approx. 0.8 mSv for both days combined (2 × 30 MBq × 0.013 mSv / MBq = 0.78 mSv), half if the patient only participates on the first day of the trial. For the average person, this equates to an increased risk of stochastic injury of 0.004% (0.002% when participating on the first day of the trial only). As our patients are among the oldest subjects, the risk of stochastic injury is reduced by a factor of 5-10 (cf. Guidelines on the use of ionizing radiation in health science experiments, Appendix 2, 2011, NVK).

Subcutaneous administration considerations: The above figures were for intravenous administration, while no immediate data are available on subcutaneous administration. Since irradiation decreases rapidly with distance (the law of distance squares), the most significant irradiation of an organ will be when the substance is in the immediate vicinity of the organ, such as in the blood supply to the organ. For the body as a whole, the irradiation will therefore mainly take place after pertechnetate has reached the bloodstream, ie. based on the above calculation. The

tissue at the injection site itself will receive a larger radiation dose than the body as a whole, but skin and muscles do not belong to the particularly radiation-sensitive parts of the body. In comparison, the limits for exposed workers are 25 times greater for skin and extremities (500 mSv / year) than for the body as a whole (20 mSv / year). Ref: Executive Order no. 669 on ionizing radiation and radiation protection (Radiation Protection Executive Order), appendix 1. https://www.retsinformation.dk/Forms/R0710.aspx?id=209405

7. Extraction of new biological material or collection of biological material from already existing biobank

During the trial, blood is continuously drawn from the patients. We will draw blood after 5, 10, 15, 30, 45, 60, 75, 90, 105, 120, 150, 180 min. after starting the infusion. We will take 5 ml at a time. We will measure activity in the blood drawn the same day (or possibly the following day) and then destroy it. The withdrawing blood is used to assess how quickly the infused fluid is found in the bloodstream. No blood or other biological material is stored in a biobank.

When participating on the second day of the trial, the patients will have blood samples taken to measure CRP, Hb, leu, Na, K, Glu, Osml, Krea, Karb, Alb before the trial is started. These will be analyzed as usual. The same blood samples will be taken from the patients' medical records in connection with the first examination, so there is no need to take them.

8. Information from patient records

The participants attending doctor will assess inpatients and discuss with the trial supervisor whether the patient meets inclusion or exclusion criteria. Relevant candidates will be contacted by the person responsible for the experiment, and we will find a time when it is appropriate for them and their relatives to review information regarding the experiment and consent statement. If patients wanted to participate, the following information will be extracted from their medical record: Age, gender, reason for admission, diagnosis list, current medication, blood pressure, heart rate, saturation, respiratory rate, blood tests (CRP, Hb, leu, Na, K, Glu, Osml , Krea, Karb, Alb). As a result of informed consent, the person responsible for the trial will be able to obtain information directly from the patient's medical record. This information may be disclosed to the relevant regulatory authority as part of quality control and or monitoring.

No information from the patients' medical records will be passed on to the person responsible for the trial before informed consent has been given, however, the person responsible for the trial can, if in doubt as to whether the patients meet exclusion criteria, assess this using medical records.

9. Processing of personal data in the project

All data will be stored securely in REDCap, and the Data Protection Ordinance and Act are complied with, cf. the Committee Act, section 20, subsection. 1, no. 4. The project has been applied for and approved by the Danish Data Protection Agency through Region North Jutland's umbrella application.

After the experiment, data will be stored according to the rules of the "Danish Code of Conduct for Research Integrity". Original data will possibly be shared in anonymised form with other researchers

after contact with the person responsible for the experiment if the purpose is found relevant, e.g. control of results, statistical method or new processing of data.

10. Economy

The project is initiated and sponsored by the Department of Geriatrics, Aalborg University Hospital. In addition, there are increased grants for the project from the Department of Endocrinology and the Department of Nuclear Medicine in the form of material and staff time.

11. Possibly. remuneration and / or other benefits to the subjects

Subjects will not receive remuneration.

12. Recruitment of subjects and informed consent

The participants attending doctor will assess whether some of the hospitalized patients are relevant for inclusion in the project. These will be contacted by the person responsible for the experiment, given written participant information and together find a time where they and their relatives (if they want them to participate) can meet in a meeting room. Here they will receive oral participant information from the person responsible for the experiment. Subsequently, they will have 24-48 hours of reflection time before they possibly sign the consent form.

Participant will at any time be able to withdraw their consent, without this will have consequences for their further processing.

Once patients are discharged from the hospital, we will contact them 3-6 weeks later to find time for examination number 2.

13. Publication of results

Attempts will be made to publish the result in relevant journals, regardless of whether the result is positive, negative or inconclusive. Furthermore, the trial will be registered on clinicaltrials.gov or other official registration site where the results will be published if they cannot be published in a journal.

14. Ethics section of science

Subcutaneous fluid administration is an infusion method with little risk of side effects and the insertion of the needle is without particular inconvenience to the patients. The radiation-related risk is small, especially when the age of the included patients is taken into account. We therefore assess that the overall risk for the included patients is small and even though there is no health benefit for the included patients, we assess that the overall scientific and future health benefit justifies the risk and the hassle.

15. Information on compensation scheme

The project is covered by The Patient Compensation.

1. Thomas DR, Cote TR, Lawhorne L, et al. Understanding Clinical Dehydration and Its Treatment. *J Am Med Dir Assoc* . 2008; 9 (5): 292-301.

2. Noriega OD, Yarlequé León SN. Antibiotics by Subcutaneous Route: A Safe and Efficient Alternative. *J Am Med Dir Assoc* . 2018; 19 (6): 553-554.
3. Lipschitz S, Campbell AJ, Roberts MS, et al. Subcutaneous fluid administration in elderly subjects: validation of an under-used technique. *J Am Geriatr Soc* . 1991; 39 (1): 6-9.
4. STONE PW, MILLER WB. Mobilization of radioactive sodium from the gastronomies muscle of the dog. *Proc Soc Exp Biol Med* . 1949; 71 (4): 529-534.
5. Cordemans C, De laet I, Van Regenmortel N, et al. Fluid management in critically ill patients: the role of extravascular lung water, abdominal hypertension, capillary leak, and fluid balance. *Ann Intensive Care* . 2012; 2 (Suppl 1): S1.
6. Roberts MS, Lipschitz S, Campbell AJ, Wanwimolruk S, McQueen EG, McQueen M. Modeling of subcutaneous absorption kinetics of infusion solutions in the elderly using technetium. *J Pharmacokinet Biopharm* . 1997; 25 (1): 1-21.
7. Caccialanza R, Constans T, Cotogni P, Zaloga GP, Pontes-Arruda A. Subcutaneous Infusion of Fluids for Hydration or Nutrition: A Review. *J Parenter Enter Nutr* . 2018; 42 (2): 296-307.
8. Mattsson S, Johansson L, Leide Svegborn S, et al. ICRP Publication 128: Radiation Dose to Patients from Radiopharmaceuticals: a Compendium of Current Information Related to Frequently Used Substances. *Ann ICRP* . 2015; 44 (2_suppl): 7-321.
