# Supplementary material for: Absorption rate of subcutaneously infused fluid in ill multimorbid older patients
Source: PLoS One. 2022 Oct 10;17(10):e0275783. doi: 10.1371/journal.pone.0275783 (PMC9550057; doi:10.1371/journal.pone.0275783)
Supplement: Attachment — Submitted filename: Response to Reviewers.docx [file pone.0275783.s006.docx]

Dear Antony Bayer

Thank you for considering our manuscript for publication in PLOS ONE and for the invitation to resubmission following revision.

The manuscript has been revised, and all points, suggestions and concerns raised by your expert reviewers have been addressed. We have updated the discussion to address review 1’s request for how this study can help clinicians, however we have balance it with reviewer 3’s concern of extrapolation. We believe the manuscript has improved by accommodating the knowledgeable comments. A point-by-point response is provided responding to every comment by the editor and reviewers.

We have reviewed the reference list to ensure all references are correct, and we have been unable to find any retracted articles. We have ensured all articles that have DOIs have it included in our reference list. Finally, we have included a recent published RCT on the safety of subcutaneous hydration.

We look forward to learning of your decision.

Respectfully yours

On behalf of the coauthors,

Mathias Brix Danielsen, MD, PhD

Aalborg University Hospital

Hobrovej 18-22

9000 Aalborg

Denmark

| **Editor comments** | **Author reply** | **Location of change in the manuscript** |
| --- | --- | --- |
| 1. Please ensure that your manuscript meets PLOS ONE's style requirements, including those for file naming. The PLOS ONE style templates can be found at  <https://journals.plos.org/plosone/s/file?id=wjVg/PLOSOne_formatting_sample_main_body.pdf> and  [https://journals.plos.org/plosone/s/file?id=ba62/PLOSOne_formatting_sample_title_authors_affiliations.pdf](https://eur03.safelinks.protection.outlook.com/?url=https%3A%2F%2Fjournals.plos.org%2Fplosone%2Fs%2Ffile%3Fid%3Dba62%2FPLOSOne_formatting_sample_title_authors_affiliations.pdf&data=05%7C01%7C%7C2c919c6515b74988823e08da644e3627%7C5968b90c51a64f088b4750ffffbe2e4f%7C0%7C0%7C637932585119783573%7CUnknown%7CTWFpbGZsb3d8eyJWIjoiMC4wLjAwMDAiLCJQIjoiV2luMzIiLCJBTiI6Ik1haWwiLCJXVCI6Mn0%3D%7C3000%7C%7C%7C&sdata=xTdCrS3YRinl2fD4dc30g%2BjpNoK14jWU8nUz9GOCqo4%3D&reserved=0) | We have updated the manuscript and supporting files to ensure they meet PLOS ONE’s style requirements. |  |
| 2. Please describe in your methods section how capacity to provide consent was determined for the participants in this study. Please also state whether your ethics committee or IRB approved this consent procedure. If you did not assess capacity to consent please briefly outline why this was not necessary in this case.” | We have followed the danish legislation for obtaining informed consent for participation in a clinical trial. This legislation requires a clinical evaluation but not a formal test for cognitive ability.  We included a sentence outlining this in the manuscript. | Methods, subsection “Participants” |
| 3. Please review your reference list to ensure that it is complete and correct. If you have cited papers that have been retracted, please include the rationale for doing so in the manuscript text, or remove these references and replace them with relevant current references. Any changes to the reference list should be mentioned in the rebuttal letter that accompanies your revised manuscript. If you need to cite a retracted article, indicate the article’s retracted status in the References list and also include a citation and full reference for the retraction notice. | We have reviewed the reference list to ensure all references are correct, and we have been unable to find any retracted articles.  We have ensured that all articles with DOI's, have the DOI included in our reference list.  Finally, we have added a recently published RCT on the safety of subcutaneous hydration. |  |

**Reviewers' comments to Author:**

Reviewer #1: nice article and interesting one, I have the following points:

| **Reviewer comment** | **Author reply** | **Location of change in the manuscript** |
| --- | --- | --- |
| **Introduction**: written well and objectives were clear | Thank you for the time and effort spent reading and providing suggestions to improve the manuscript. |  |
| **Methods:**  written well and detailed |  |  |
| 1) why did only include 6 patients knowing that you will not include any control as per the initial study design? was it based on any calculations or just an arbitrary number? no details were mentioned on sample size part on how it was calculated | We agree that this is a key point. We aimed to portray the patients in our geriatric wards: elderly patients with concurrent comorbidities, poor physiological reserve, and few routes of pharmacological administration.  Recruiting among the frail hospitalized geriatric patients and obtaining informed consent was cumbersome, and the time and strain of just asking them to consider participation was clearly a burden to them.  However, this group of patients may also benefit the most from subcutaneous hydration and those who differ the most from the previous report among healthy individuals (Lipschitz, 1991).  We thus saw a justification for the present study, but we still had to balance the discomfort and strain on these frailest patients against the number of participants needed. The number of 6 included patients was based on our sample size calculation. The previous study on the subject (Lipschitz, 1991) also managed with 6 participants; as it turned out, we had sufficient data with this number.  We have updated the sample size section to include this information. | Methods, subsection “Sample size” |
| 2) did use any pump for the infusion or only gravity? | Thank you for emphasising this point. We did use an infusion pump to control the infusion rate, and this relevant information is now added to the manuscript. | Method, subsection “Study setup” |
| 3) based on what did use that infusion rate 250 ml per hour?? as in clinical practice that's very quick and I doubt anyone will use it in clinical setting unless in emergency situation | The very high infusion rate was a middle ground between the infused volume (preferred high, as in clinical practice) and the study duration feasible for the multimorbid older patients. All included patients were exhausted by the end of the second hour after the completion of the infusion. They all requested to be transferred back to the ward by then. This is in keeping with our inclusion of the (intended) very frail target group.  We have included a sentence in the method section to clarify our reason for the high infusion rate.  Furthermore, we have rewritten the section in the discussion where we extrapolate our data to a slower infusion rate and larger volume. However, extrapolation should always be interpreted with caution. | Method, subsection “Study setup”  Discussions, third paragraph |
| **Results**: written well and detailed | Thank you. |  |
| **Discussion**: written well and detailed I think you should add how your study results can help physicians on treating similar patients other than what we already knows | Thank you for this suggestion extending our intention with this study. We have added a section in the discussion to clarify how our results can help physicians. | Discussion, second to last paragraph. |

Reviewer #2:

A small exploratory research study (n=6) was conducted which aimed to describe the absorption rate of subcutaneous infused fluid in a select group of patients. Upon completion of the infusion (at 60 minutes), 53% of the infused fluid was absorbed; an hour later it was 88%. The absorption rate was 127 ml/h immediately after the completion of the infusion.

Response:

*Thank you for the time and effort put into reading our manuscript and for the useful comments to improve our report.*

Minor revisions:

| **Reviewer comment** | **Author comment** | **Location of change in the manuscript** |
| --- | --- | --- |
| 1) Abstract: Provide 95% confidence intervals for the 53%, 88% and the rate of 127 ml/h. | We have included the 95% confidence interval for both the infused fluid per cent at 60 mins and 120 mins in the abstract. | Abstract, results section |
| 2) Line 151: A beta of 0.2 corresponds to a power of 80%. Perhaps the beta is misspecified. Indicate the statistical testing method which achieves the specified alpha and power. | You are correct that we have a misspecified beta. We used a power of 80%, which has been corrected in the text. Thank you. | Methods section, subsection “Sample size” |
| 3) Considering that the sample size is small and the distribution of the data from small sample sizes cannot be shown to be normal, it is standard practice to summarize these types of data using median, first and third quartiles. | While we agree that median and quartiles are relevant when data are not normally distributed, the benefit for small datasets is unclear to us. Our study has six data points, so the first quartile would be midway between datapoint #2 and #3, and the third quartile between #4 and #5. The graphs for the patients do not indicate that any of them were outliers, which indicates that the mean and standard deviation will be representative.  The justification has been added, and we hope this point is acceptable for supporting the presentation of the data in our report. | Methods section, subsection “Statistical analysis and calculations” |
| 4) Table 1: Specify the percent male. | We have added the percentages in table 1 as recommended. | Table 1 |
| 5) Line 263: Specify the statistical testing method used to estimate the p-value for the significant correlation. Perhaps a graph might illustrate the correlation better than specifying the p-value or providing an effect size. | We have included a paragraph describing how this p-value was estimated. However, we believe the effect size estimate is too difficult to interpret meaningfully to be included. From clinical experience, there is a minimum serum albumin level where SC hydration is no longer advisable. The reason to include a p-value without the size estimate is to state that our results corroborate this clinical experience. | Methods section, subsection “Statistical analysis and calculations”, second to last paragraph. |

Reviewer #3:

Although it is a small study, the sample size is sufficient to demonstrate the ability of the subcutaneous tissue to absorb liquids,therefore, it is an interesting and useful study that is a little closer to the reality that many of us see in daily clinical practice: elderly patients with concurrent comorbidities, poor physiological reserve, and few routes of pharmacological administration.

The introduction encompasses the problem efficiently, the methodology and results are correct and well developed.The main limitation is the administered volume, which is scarce, and it only reflects the initial absorption capacity of the subcutaneous tissue. As fluid is infused through this route, accumulation is progressive and the residual volume itself limits absorption capacity, reducing absorption speed and tolerance. Although it is described in the limitations, it is very important because it does not allow us to extrapolate these results to clinical where infusion of larger volumes is often necessary.

However, I consider a good, interesting, and practical study that allows us to take another step towards the reality of many patients, so I recommend it to be considered for publication

Response:

*Thank you for the time and effort put into reading our manuscript. We appreciate the knowledgeable and useful comments to improve our report.*

| **Reviewer comment** | **Author comment** | **Location of change in the manuscript** |
| --- | --- | --- |
| Another limitation of the study is found in the type of patients included, since the limited variety of conditions makes it impossible to assess other aspects that may influence absorption apart from hypoalbuminemia or hypotension. | We very much agree with your mindset. Many future substudies are needed to extend our knowledge on absorption in patients with other conditions.  i.e. is there a lower limit for s-albumin where SC hydration is no longer feasible? |  |
| It would be interesting if they described a little more what solution they are referring to, when speaking of isotonic saline, it is intuited that it is the "normal" 0,9% saline, but any balanced crystalloid could respond to that description. This could be relevant because there are differences in local tolerance between crystalloids and possibly altered absorption speed (although theoretically of little relevance, it is in these patients where every detail counts). | Thank you for this comment. We agree that such details are relevant to our most frail patients, and the infusion solution should be described unambiguously. We have included a clarification. | Method, subsection “Study setup”. |
