# Supplementary material for: Absorption rate of subcutaneously infused fluid in ill multimorbid older patients
Source: PLoS One. 2022 Oct 10;17(10):e0275783. doi: 10.1371/journal.pone.0275783 (PMC9550057; doi:10.1371/journal.pone.0275783)
Supplement: Attachment — Submitted filename: Response to Reviewers.docx [file pone.0275783.s007.docx]

Denmark, September 2022

Dear Antony Bayer

Again thank you for considering our manuscript for publication in PLOS ONE and thank you for the very fast reassessment of our manuscript.

We have review the manuscript and revised according to the comments from the editor and reviewer 2.

We look forward to learning of your decision.

Respectfully yours

On behalf of the coauthors,

Mathias Brix Danielsen, MD, PhD

Aalborg University Hospital

Hobrovej 18-22

9000 Aalborg

Denmark

| **Editor comments** | **Author reply** | **Location of change in the manuscript** |
| --- | --- | --- |
| Please can you ensure also that you use consistent terminology to describe the patients you studied. For example, your title describes them as “ill multimorbid…”, then the background of your abstract refers to “geriatric patients”, the methods to “frail, ill octogenarians with comorbidities” and the conclusion to simply “octogenarians”. There is similar variation throughout the text. They do seem to have been ill and multimorbid and on a geriatric ward, but I am not sure about all being frail or octogenarians? | It is indeed correct that they were ill, multimorbid and on our geriatric ward. We used different wordings to describe our included patients to support variation in the text. We can see how this may create confusion and some of the words were not fully correct. We have aligned the description of our included patients throughout the text. | Throughout the manuscript |
| “Data” are plural – please correct as necessary e.g., line 210 and 294 (and maybe others). | Thank you for bringing this to our attention. All instances where data are described have been inspected and corrected were needed. | Line 211, 299 |
| Line 276 – …thyroid gland AT 58 minutes… | The sentence at line 276 has been rephrased to support readability, while the verb is kept in singular as the noun is “the plateau value”. | Line 300 |

**Reviewers' comments to Author:**

Minor revisions:

| **Reviewer comment** | **Author comment** | **Location of change in the manuscript** |
| --- | --- | --- |
| 1- Graphs: Percentage is misspelled. | Thank you. This has been corrected. |  |
| 2- Table 1: Percent is misspelled | Thank you. This has been corrected. |  |
| 3- State the statistical methods used to estimate the 95% confidence intervals. | We are not quite sure if you would like us to include the standard method for calculating confidence interval “95% CI = mean ± (SD/SQR(n)”. However, we think you might be referring to the 95% CI for the half-times. We have added a sentence to describe that these CI’s were derived from the half-time for the individual patients. | Line 205-206. |
